# Adiposity and adipogenic gene expression in four different muscles in beef cattle

**DOI:** 10.1371/journal.pone.0179604

**Published:** 2017-06-30

**Authors:** Lara Martínez del Pino, Ana Arana, Leopoldo Alfonso, José Antonio Mendizábal, Beatriz Soret

**Affiliations:** Escuela Superior de Ingenieros Agrónomos, Departamento de Producción Agraria, Universidad Pública de Navarra, Pamplona, Spain; Wageningen UR Livestock Research, NETHERLANDS

## Abstract

Anatomical site and divergent functionalities of muscles can be related to differences in IMF content, metabolism and adipogenic gene expression. Then, potential differences in different muscles in beef cattle were studied. As a second objective, the main sources of experimental variability associated to RT-qPCR results were analyzed following a nested design in order to implement appropriate experimental designs minimizing gene expression variability. To perform the study *Longissimus thoracis (LT)*, *Semitendinosus (SM)*, *Masseter (MS)*, *Sternomandibularis (ST)* and subcutaneous adipose tissue (SAT) samples of Pirenaica young bulls (n = 4) were collected for IMF, collagen and protein quantification, analysis of adipocyte size distribution and gene expression (*PPARG*, *CEBPA*, *FAPB4* and *WNT10B*). A greater IMF content was observed in *MS* and *SM* muscles, which had a bimodal adipocyte size distribution while it was unimodal in the muscles *LT* and *ST*. This suggest that the different IMF accretion in the muscles studied might be related to different rates of hyperplasia and hypertrophy and that IMF might develop later in *LT* and *ST* muscles. The former differences were not mirrored by the expression of the genes analyzed, which might be related to the different contribution of mature and non-mature adipocytes to the total gene expression. When comparing IMF and SAT gene expression, late and early developing tissues respectively, expression of *PPARG*, *CEBPA* and *FABP4* was higher in the SAT, in agreement with bigger cell size and numbers. The variability study indicates that the analytical factors that add higher variability to the gene expression are the sampling and RT and therefore, it would be appropriate to include those replicates in the design of future experiments. Based on the results, the use of *MS* and *SM* muscles could allow less expensive experimental designs and bigger sample size that could permit the detection of lower relevant differences in gene expression.

## Introduction

It is well established that muscles display differences in muscle fibre type, proteolytic activity, connective tissue and intramuscular fat (IMF) percentage, attributes that are related to the molecular, metabolic, structural, and contractile properties of the muscle [1, 2]. The former characteristics have a deep influence on meat quality traits as well [3]. IMF plays an essential role in the determination of meat quality and it is acknowledged as an economically important feature [4, 5]. In some cases, quality of meat can be compromised by the low tendency of some cattle populations to accumulate IMF. This is the case of Pirenaica breed for example, which is widely used in northern Spain and is highly appreciated for its value as a genetic resource and its contribution to the maintenance of local beef production systems [6]. Different approaches have been considered with the objective of increasing Pirenaica IMF content, as extending the fattening period from 12 months of age (the usual endpoint) to 18 and increasing the feed energy density, but neither seem to have a significant effect on the amount of IMF in the *longissimus* muscle as shown in a previous and independent study [6]. Then, deepening the knowledge of IMF accretion mechanisms would be important to find appropriate strategies to increase the value of the meat produced in these systems.

Most of the studies performed in cattle up to date have focused on evaluating the influence of external factors like breed, age and diet in the *Longissimus thoracis* muscle. Nevertheless, studying just the *Longissimus* muscle might be limiting as the different anatomical regions and divergent functionalities of muscles can be related to differences in fat accretion and metabolism [4, 7]. This in turn could be associated to differential expression of key genes involved in the IMF deposition and development. Furthermore, some experimental designs that require a high sampling size might not be affordable due to the high commercial value of *Longissimus* muscle. Therefore, it would be of interest to analyze the potential differences between different muscles when studying the mechanisms that regulate IMF accretion in cattle.

Real time reverse transcription PCR (RT-qPCR) allows quantification of the changes in gene expression [8–10] and can be a useful technique to gain knowledge about the IMF deposition process. However, a wide range of analytical situations, apart from the animal, the tissue or the experimental treatment assayed, may affect gene expression [11]. In order to improve the experimental designs and to achieve a more accurate analysis of the experimental hypothesis it is of interest to analyze the different analytical factors that affect RT-qPCR results [12]

The first objective of this work was to assess the possible differences in fat, collagen and protein content as well as in adipocyte size distribution of different muscles and to determine if the expression of some key adipogenic genes was muscle and depot dependent. The muscles selected for the study were: *Longissimus thoracis* (*LT*), *Semitendinosus* (*ST*), *Masseter* (*MS*), and *Sternomandibularis* (*SM*). The criteria for muscle selection were the dominant metabolism, being either predominantly glycolytic (*LT* and *ST*) [13–15] or predominantly oxidative (*MS* and *SM*) [16–18], and the commercial value [19, 20]. In order to study the effect of depot on the adipogenic gene expression, and to take as reference a widely studied tissue, subcutaneous adipose tissue (SAT) [21, 22] from the *longissimus* area was also considered. The second aim was to study the main sources of experimental variability associated with gene expression in order to implement appropriate experimental designs that could allow to minimize the experimental variability.

## Material and methods

### Ethical approval

Animal care, handling and experimental procedures complied with international guidelines (European Union procedures on animal experimentation-Directive 2010/63/EU) that regulate the protection of animals used for scientific purposes [23], which define that for experiments carried out under standard production conditions no approval from an ethic committee is required. The bulls were born and housed in commercial farms and the production system was the usual for Pirenaica cattle in Navarra (Spain): the calves were with their mothers up to six months of age and were offered a standard concentrate growing diet and cereal straw, both *ad libitum*. From then and up to 12 months, the commercial endpoint, the young bulls were kept in large partially shaded pens and fed a standard fattening diet based on concentrate and cereal straw, both *ad libitum*.

On the day of the sampling, the bulls were transported to a commercial abattoir (“La Protectora”) in Pamplona (Spain) which had already issued the permission to undertake the sampling. Animal care and handling in the farm and during transportation followed European guidelines [24, 25]. The slaughter was performed by using stunning methods (non-penetrative captive bolt device immediately followed by bleeding) following the European Union regulations that regulate the protection of animals at the time of killing (Council Regulation, EC, No 1099/2009) [26]. Therefore, animals included in this experiment were subjected to the same welfare conditions as production animals in farms and abattoir and all efforts were made to minimize suffering.

The experimental protocol was approved by the Committee on Ethics, Animal Experimentation and Biosecurity of the Public University of Navarre (permit number PI 013/14).

### Animals and sample collection

Pirenaica young bulls (n = 4) were slaughtered at an age of 11.9 ± 0.66 months; the average carcass weight was 324.0 ± 17.66 kg. Immediately after slaughter, samples of muscles *LT* at the 10^th^ rib, *ST*, *MS*, and *SM* on the left side of the carcass were collected, trimmed of external fat and connective tissue and divided into portions for quantification of the chemical traits, adipocyte size and RNA isolation. SAT at the 10^th^ rib of the left carcass side was also collected and divided in portions for adipocyte size analysis and RNA isolation.

The different portions of the samples were divided as follows: the sub-samples taken for tissue chemical characterization were placed in ice and stored at -20°C until use; sub-samples for adipocyte size determination were placed in test tubes and kept at 39°C in Tyrode solution (0.15 M NaCl, 6 mM KCL, 2 mM CaCL_2_, 6 mM C_6_H_12_O_6_, 2 mM NaHCO_3_, pH 7.62), and the sub-samples intended for RNA extraction were deep frozen in liquid nitrogen and stored at -80°C until use.

### Experimental design

**Fig 1:**
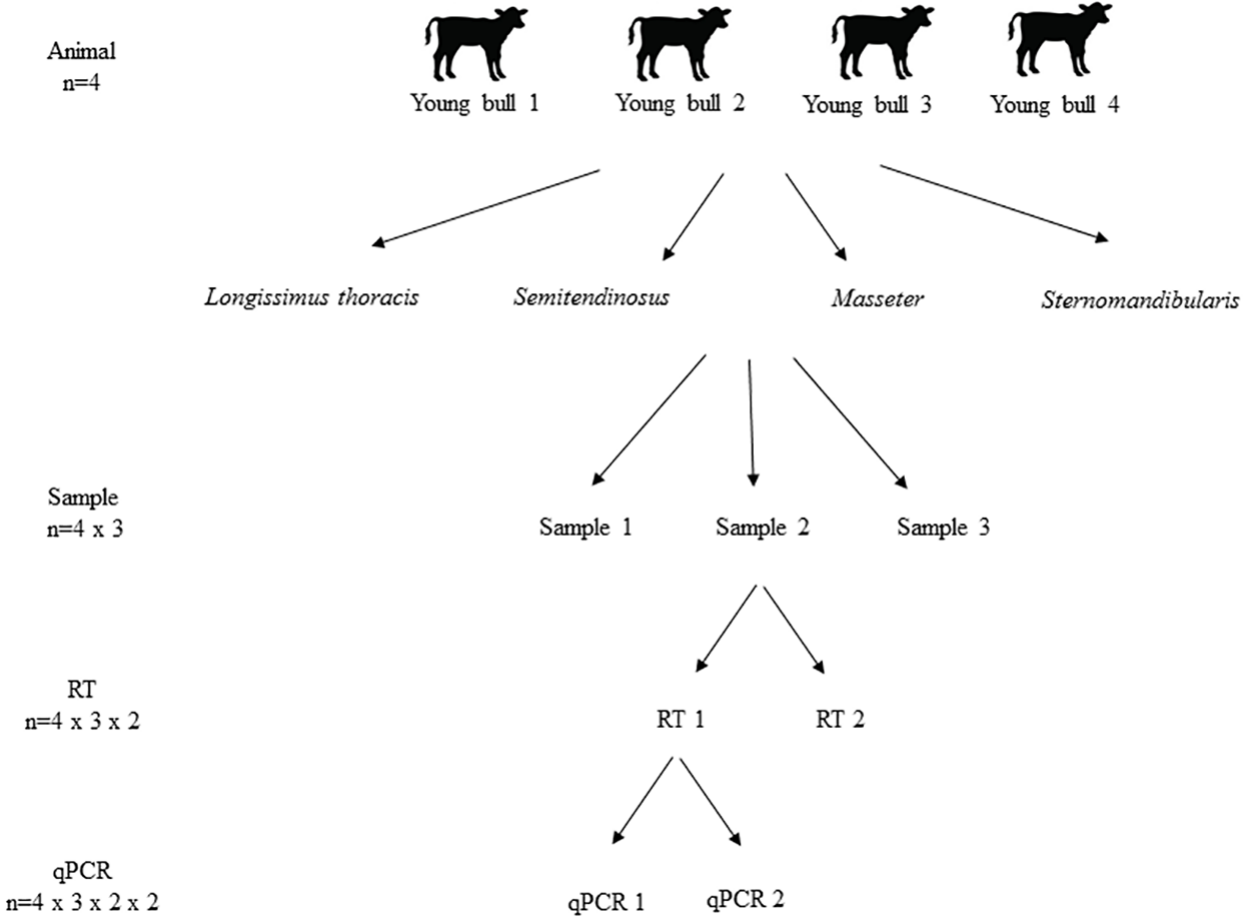
Experimental design for gene expression variability analysis. Three RNA replicates were extracted from each tissue sample (four muscles for each animal), two reverse transcription (RT) replicates were performed for each extract and two qPCR replicates were performed for each RT, following the model: [(nA = 4) x (nS = 3) x (nRT = 2) x (nqPCR = 2)], where A = animal and S = sample, producing a total of 48 Cq values per tissue and gene.

A nested design for the following steps of the analytical process: sampling (RNA extraction), reverse transcription (RT) and quantitative polymerase chain reaction (qPCR) was considered. For RNA extraction, three sub-samples (RNA-samples) were taken from the muscles at slaughter. Then, as described in Fig 1, from each RNA sample two RT were performed and two qPCR replicates for each RT were run. The laboratory analyses (RNA extraction, RT and qPCR) were completely randomized in order to minimize the experimental variability. This design allows the quantification of the variability due to RNA extraction, RT and qPCR in the gene expression of the adipogenic genes of interest.

### Chemical traits

The amount of chemical fat was determined by the Soxhlet method (International Organization for Standardization-ISO 1443, 1973). The Kjeldahl method was used to determine the protein percentage [27]. Total collagen was quantified by measuring the total hydroxyproline according to the method described by Bergmann-Loxley [28].

### Adipocyte size analysis

Adipocytes were extracted from the four muscles and the SAT following the technique described by Robdell [29] slightly modified to extract intramuscular adipocytes [6, 30]. As indicated, the samples were transported to the laboratory in the Tyrode solution at 39°C within 20 min after slaughter. Then, using a thermal block to maintain the temperature at 39°C, connective tissue and blood vessels were removed by using scalpel and scissors and the tissues were minced and rinsed with saline serum. The samples were then digested by incubation for 90 min at 39°C in a flask with 5 ml of Medium 199 (Gibco 31150–022, Waltham, MA, USA) (pH: 7.0 to 7.4), 200 mg of bovine serum albumin (Sigma-Aldrich, Madrid, Spain) and 5 or 10 mg of collagenase type II (Sigma-Aldrich, Madrid, Spain) for SAT and muscle respectively. After the collagenase digestion, adipocyte solutions were filtered through a 850 μm mesh size to remove tissue debris. Adipocyte size diameter was determined by measuring at least 200 cells per sample by image analysis [31]. For that purpose, the cells were examined microscopically using an Olympus BH.2 microscope (Olympus Optical Co., Europa Combth Postfact 104908; 20034 Hamburg). Measurement of adipocyte size was performed using a program for the acquirement and storage of the images as well as for the processing of data (Image-Pro Plus 5.1, Media Cybernetics, Inc. Silver Spring MD 20910). It was established that adipocytes should meet a shape criteria, so adipocytes have a shape factor of 0.8–1 (shape factor of 0 indicating a straight line and 1 a perfect circle).

### RNA Isolation and RT-qPCR

RNA from the four muscles was isolated using Trizol^®^ reagent (Invitrogen, Carlsbad, CA, USA) and GenElute Mammalian Total RNA Miniprep kit (Sigma-Aldrich Química, Madrid, Spain) according to the manufacturer´s instructions. RNA was isolated from SAT samples using the RNeasy Lipid Tissue Kit (Qiagen, Hilden, Germany) also following manufacturer´s recommendations. Concentration and purity of the RNA was assessed by using a NanoDrop 2000 spectrophotometer (Thermo Scientific, Madrid, Spain) by optical density quantification at 260 and 280 nm. A total of 750 ng of RNA was treated with DNase using RQ1 RNases-Free DNase (Promega Corporation, Madison, WI, USA) and 500 ng of RNA were used to obtain singled stranded cDNA using PrimeScript RT Reagent (Takara, Japan) following manufacturer´s instructions (S1 File).

The expression of genes related to adipogenesis: *peroxisome proliferator-activated receptor γ* (*PPARG*), *CCAAT/enhancer-binding protein α* (*CEBPA*), *fatty acid binding protein 4* (*FABP4*) and *wingless-type MMTV integration site family 10B* (*WNT10B*) (S1 Table), was analysed using a FX96 Touch^™^ Real-Time PCR Detection System (Bio-Rad, Munich, Germany). Briefly, qPCR was conducted in a total reaction volume of 10 μl with 5 μl SYBR Premix Ex Taq (Takara, Japan), 0.2 μl of 10 μM primers each, 3 μl of cDNA (dilution 1:5), and 1.6 μl DNase-RNase Free H_2_O.

Thermal cycling conditions were as follows: 95°C for 30 min, followed by 40 cycles at 95°C for 5 s and 60°C for 30 s, followed by amplicon dissociation at 65°C for 5 s and 95°C for 5 s. Dissociation curves were examined for the presence of a single RT-qPCR product (S1 File).

*β-actin (ACTB)* and *Topoisomerase II-beta* (*TOP2B)* (S1 Table) were used as reference genes, based on the stability values obtained in previous experiments. The expression of the reference genes was analyzed following the same methodology previously described. The efficiency of all primers ranged from 1.87 to 2.00 and R^2^ were close to 0.99 for all the genes (S2 Table).

### Statistical methods

#### Chemical traits

In order to determine if there were differences in chemical traits between tissues, data were analyzed by one-way analysis of the variance (ANOVA).

#### Adipocyte size

Adipocyte size distribution was studied with the AdipSD software [32]. This software allows to determine if the adipocyte size distribution is unimodal or bimodal using the bimodality coefficient (BC) (SAS Institute Inc., Cary, NC, USA) and Dip [33] tests. BC test is based on the number of observations, sample bias and excess of kurtosis. In a unimodal distribution, the BC value should be 0.555 or less. Higher BC values signify a bimodal distribution of the adipocytes; however, it may also indicate a strongly skewed unimodal distribution [32]. Dip test is described as the maximum difference between the empirical distribution function, and the unimodal distribution function that minimizes that maximum difference. When *P*-value in the Dip test is less than 0.5, the unimodality hypothesis is not rejected. Contrary to BC, Dip is not implemented in most commonly used statistical packages, but in case of lack of convergence between the two tests, Dip has been considered the most appropriate measure [34]

#### Gene expression

Gene expression was analyzed for each marker using the MIXED procedure (SAS 9.2, SAS Institute Inc., Cary, NC, USA), which is appropriate for completely randomized designs [11]. To account for differences in amplification efficiencies (E) between the target and reference genes, Cq data were log_2_ transformed using the expression log_2_ (E ^−Cq^).

The tissue, i.e. the four muscles studied and SAT, and gene combination were considered according to the following model:

$$y_{ijklmn}=\mu +T_{i}\times G_{j}+A_{k}+S_{l}(A_{k})+R_{m}(S_{l}(A_{k}))+e_{ijklmn}$$

Where *y*_*ijklmn*_ is the individual response expressed as log_2_ (E ^−Cq^), μ is the mean, *T*_*i*_ is the fixed effect of the tissue analyzed (i = 5; *LT*, *ST*, *MS*, *SM*, SAT), *G*_*j*_ the fixed effect of the gene studied (j = 3; target gene, reference genes), *A*_*k*_ is the random effect of the k^*th*^ animal (*A ~ N*(*0*,*σ*^*2*^_*A*_)), *S*_*l*_*(A*_*k*_*)* is the random effect of the l^*th*^ sample taken from the k^*th*^ animal *(S ~ N(0*,*σ*^*2*^_*S*_*))*, *R*_*m*_*(S*_*l*_*(A*_*k*_*))* is the random effect of the m^*th*^ RT reaction of l^*th*^ sample from k^*th*^ animal *(R ~ N(0*,*σ*^*2*^_*R*_*))*, and *e*_*ijklmn*_ is the residual random effect of each qPCR (*e ~ N*(0,*σ*^*2*^_*e*_)).

Different sub-models were defined in order to consider the variance heterogeneity between gene and tissue for the following random factors: animal (*A ~ N*(*0*,*σ*^*2*^_*Aij*_)), sample (*S ~ N*(*0*,*σ*^*2*^_*Sij*_)), RT (*R ~ N*(*0*,*σ*^*2*^_*Rij*_)) or qPCR (*e ~ N*(0,*σ*^*2*^_*eij*_)). The sub-models were analyzed using the MIXED and, those that always converged to a solution, compared using corrected Akaike's and Schwarz's Bayesian information criterion values. The sub-model that presented heterogeneous variance for Sample and RT effects was chosen. Different contrasts were defined to estimate gene expression differences (DIF) between tissues, as the minus the difference between normalized Cq values:

$$DIF=\left(y_{T_{1}G_{t}}-\overset{2}{\underset{i=1}{\sum }}y_{T_{1}G_{r_{i}}}\right)-\left(y_{T_{2}G_{t}}-\overset{2}{\underset{i=1}{\sum }}y_{T_{2}G_{r_{i}}}\right)$$

Where, G_t_: target gene; G_r_: reference genes.

#### Variability analysis

The optimal experimental design requires to know the sources of error throughout sample processing. This question was addressed by a variance decomposition analysis to study the contribution of factors that affect the total variance of the experiment. The RT-qPCR results were analyzed by the NESTED procedure (SAS 9.2, SAS Institute Inc., Cary, NC, USA) following the experimental design defined in Fig 1.

For each tissue (*i*) and each gen (*j*), data were analyzed using the following model:

$$y_{ijklmn}=\mu +A_{k}+S_{l}(A_{k})+R_{m}(S_{l}(A_{k}))+e_{ijklmn}$$

Where, *y*_*ijklmn*_ is the individual response expressed as Cq. *A*_*k*_ is the random effect of the k^*th*^ animal (*A ~ N*(*0*,*σ*^*2*^_*A*_)), *S*_*l*_*(A*_*k*_*)* is the random effect of the l^*th*^ sample taken from the k^*th*^ animal *(S ~ N(0*,*σ*^*2*^_*S*_*))*, *R*_*m*_*(S*_*l*_*(A*_*k*_*))* is the random effect of the m^*th*^ RT reaction of l^*th*^ sample from k^*th*^ animal *(R ~ N(0*,*σ*^*2*^_*R*_*))*, and *e*_*ijklmn*_ is the residual random effect of each qPCR (*e ~ N*(0,*σ*^*2*^_*e*_)).

The variance contributions of the processing steps were estimated as follows:

$${\hat{\sigma }}_{ct}^{2}=\;{\hat{\sigma }}_{k}^{2}+{\hat{\sigma }}_{l}^{2}+{\hat{\sigma }}_{m}^{2}+{\hat{\sigma }}_{n}^{2}$$

Variance contributions were also expressed as percentages:

$$Variance\;contribution=100\;\times \;{\hat{\sigma }}_{x}^{2}/{\hat{\sigma }}_{Ct}^{2}$$

Where *x* = *k*, *l*, *m* or *n* (animal, sample, RT and qPCR respectively).

## Results

### Chemical traits and adipocyte size analysis

Chemical fat content was higher in *MS* and *SM* compared to *LT* and *ST* muscles (*P <* 0.05) but no statistically significant differences were found in the protein content among muscles (Table 1). Total collagen content was also similar between muscles (data not shown).

**Table 1:** Mean values and mean standard errors for the chemical traits measured in *Longissimus thoracis*, *Semitendinosus*, *Masseter*, and *Sternomandibularis* muscles.

Item	*LT*^1^	*ST*	*MS*	*SM*	SEM	*P*-value
Fat, %	1.30^b^	1.34^b^	3.22^a^	3.15^a^	0.30	0.005
Protein, %	22.16	21.59	20.75	21.02	0.39	0.112

^1^*LT* = *Longissimus thoracis*, *ST* = *Semitendinosus*; *MS* = *Masseter*; and *SM* = *Sternomandibularis*

^a,b^ Means with different superscripts within a row are different (*P <* 0.05).

**Fig 2:**
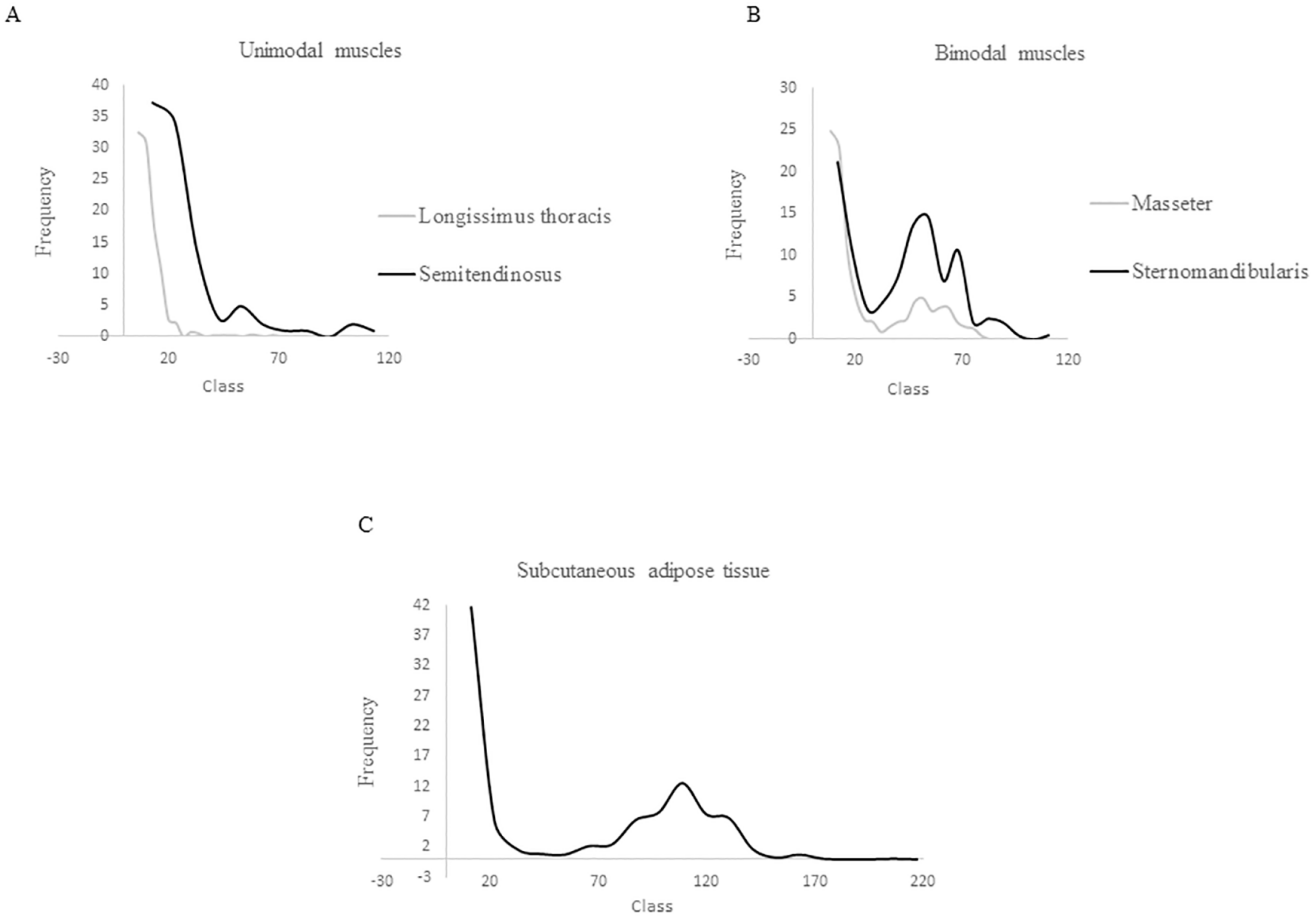
Adipocyte size distribution of the tissues studied. A) adipocyte size distribution in the muscles *Longissimus thoracis* and *Semitendinosus*; B) adipocyte size distribution in the muscles *Masseter* and *Sternomandibularis*; C) adipocyte size distribution in the subcutaneous adipose tissue.

According to the unimodality-bimodality study based on BC and Dip tests, muscles presented different adipocyte size distributions. Adipocytes from *LT* and *ST* muscles had a unimodal distribution while the adipocytes from *MS* and *SM* muscles showed a bimodal distribution. The cell size distribution for adipocytes obtained from the SAT was also bimodal (Fig 2).

It was observed that the minimum, mean, mode (*P* < 0.05) and median (*P* ≤ 0.001) of the adipocytes were significantly smaller in *LT* than in *ST* muscles (Table 2), both with unimodal adipocyte size distribution. Regarding the tissues that showed a bimodal adipocyte size distribution (*MS*, *SM*, and SAT), the minimum adipocyte size was significantly lower in *SAT* compared to *SM* muscle (*P <* 0.05) (Table 3) while the maximum adipocyte size was similar between both muscles and greater in SAT (*P <* 0.05). The first mode was significantly lower in *MS* compared to *SM* muscle (*P <* 0.05) and was similar between SAT and both muscles. The second mode was significantly lower in *MS* and *SM* muscles than in SAT (*P <* 0.001). On the other hand, the nadir, the percentage above the nadir and the small/large ratio, showed no significant differences between tissues.

**Table 2:** Descriptive parameters for adipocyte size distribution in *Longissimus thoracis and Semitendinosus* muscles.

Unimodal distribution				
Item	*LT*^1^	*ST*	SEM	*P*-value
Minimum, μm	5.68	9.80	0.63	0.005
Maximum, μm	48.78	66.22	13.26	0.404
Median, μm	10.11	21.40	0.82	0.001
Mean, μm	13.25	26.79	2.63	0.013
Mode, μm	10.35	17.45	1.51	0.019

^1^*LT* = *Longissimus thoracis*, *ST* = *Semitendinosus*

**Table 3:** Descriptive parameters for adipocyte size distribution in *Masseter*, *Sternomandibularis* muscles, and subcutaneous adipose tissue.

Bimodal distribution					
Item	*MS*^1^	*SM*	SAT	SEM	*P*-value
Minimum, μm	7.25^a^^b^	10.29^a^	6.89^b^	0.79	0.026
Maximum, μm	69.5^b^	89.62^b^	163.61^a^	9.73	0.001
Nadir, μm	27.07	34.97	38.90	3.15	0.126
% above the nadir	35.62	55.87	48.55	6.25	0.148
Small/large	1.97	0.93	1.12	0.30	0.091
First mode, μm	10.68^b^	18.39^a^	12.61^a^^b^	1.58	0.029
Second mode, μm	55.26^b^	59.04^b^	113.95^a^	4.96	0.000

^1^*MS* = *Masseter*; *SM* = *Sternomandibularis*; SAT = subcutaneous adipose tissue

^a,b^ Means with different superscripts within a row are different (*P <* 0.05).

### Gene expression

To determine if there were differences in the gene expression between tissues, a MIXED model was used to compare the different tissues one by one, as described previously.

No differences were found between muscles for *PPARG*, *CEBPA* and *WNT10B* expression (Table 4). The expression of *FABP4* was significantly higher in *LT* muscle compared to *MS* (*P <* 0.05) and *SM* (*P <* 0.001).

**Table 4:** Differences in normalized expression values between muscles *Longissimus thoracis*, *Semitendinosus*, *Masseter* and *Sternomandibularis*.

Gene	Contrast	DIF^2^	SEM	*P*-value
*PPARG* ^1^	*LT-MS*^3^	0.53	0.42	0.215
	*LT-ST*	0.43	0.78	0.584
	*LT-SM*	1.01	0.53	0.061
	*MS-ST*	-0.09	0.78	0.907
	*MS-SM*	0.49	0.53	0.358
	*ST-SM*	0.58	0.85	0.501
*CEBPA*	*LT-MS*	0.42	0.46	0.361
	*LT-ST*	-0.15	0.74	0.839
	*LT-SM*	-0.03	0.45	0.939
	*MS-ST*	-0.54	0.81	0.507
	*MS-SM*	-0.45	0.49	0.363
	*ST-SM*	0.07	0.81	0.930
*FABP4*	*LT-MS*	1.36	0.57	0.020
	*LT-ST*	1.16	1.31	0.379
	*LT-SM*	2.24	0.67	0.001
	*MS-ST*	-0.19	1.25	0.879
	*MS-SM*	0.88	0.59	0.139
	*ST-SM*	1.08	1.32	0.416
*WNT10B*	*LT-MS*	-0.32	0.62	0.606
	*LT-ST*	-0.42	0.80	0.604
	*LT-SM*	-1.13	0.64	0.082
	*MS-ST*	-0.10	0.70	0.886
	*MS-SM*	-0.82	0.54	0.137
	*ST-SM*	-0.72	0.77	0.351

^1^*PPARG = peroxisome proliferator-activated receptor γ*; *CEBPA = CCAAT/enhancer-binding protein α; FABP4 = fatty acid binding protein 4; WNT10B* = wingless-*type MMTV integration site family 10B*.

^2^DIF = contrasted differences between normalised log_2_ (E ^−Cq^) values.

^3^*LT* = *Longissimus thoracis*, *ST* = *Semitendinosus*; *MS* = *Masseter*; and *SM* = *Sternomandibularis*

The expression of the genes of interest in the four muscles studied was also compared to their expression in the SAT (Table 5). *PPARG*, *CEBPA* and *FABP4* expression was significantly higher in the SAT than in the muscles (*P <* 0.01), but *WNT10B* expression did not show any differences between depots (*P >* 0.05).

**Table 5:** Differences in normalized expression values between *Longissimus thoracis*, *Semitendinosus*, *Masseter*, *Sternomandibularis*, and subcutaneous adipose tissue.

Gene	Contrast	DIF^2^	SEM	*P*-value
*PPARG* ^1^	SAT-*LT*^3^	2.07	0.52	0.000
	SAT-*MS*	2.56	0.48	0.000
	SAT-*ST*	2.51	0.76	0.002
	SAT-*SM*	3.07	0.51	0.000
*CEBPA*	SAT-*LT*	3.50	0.52	0.000
	SAT-*MS*	3.87	0.54	0.000
	SAT-*ST*	3.38	0.80	0.000
	SAT-*SM*	3.45	0.49	0.000
*FABP4*	SAT-*LT*	7.48	0.60	0.000
	SAT-*MS*	8.82	0.47	0.000
	SAT-*ST*	8.68	1.25	0.000
	SAT-*SM*	9.75	0.56	0.000
*WNT10B*	SAT-*LT*	0.51	0.56	0.368
	SAT-*MS*	0.12	0.39	0.762
	SAT-*ST*	0.11	0.66	0.664
	SAT-*SM*	-0.67	0.48	0.168

^1^ *PPARG = peroxisome proliferator-activated receptor γ*; *CEBPA = CCAAT/enhancer-binding protein α; FABP4 = fatty acid binding protein 4; WNT10B* = wingless-*type MMTV integration site family 10B*.

^2^DIF = contrasted differences between normalised log_2_ (E ^−Cq^) values.

^3^*LT* = *Longissimus thoracis*, *ST* = *Semitendinosus*; *MS* = *Masseter*; and *SM* = *Sternomandibularis*; SAT = subcutaneous adipose tissue.

### Variability analysis

**Fig 3:**
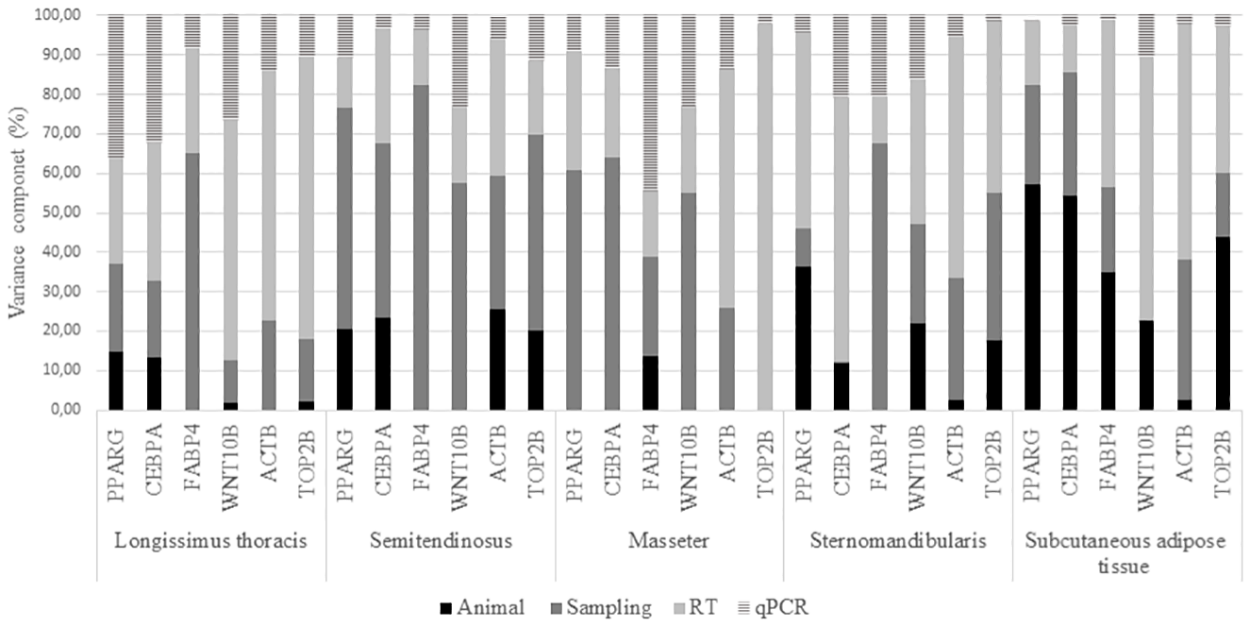
Standard deviations contributing for the sampling-processing steps expressed as percentages. *PPARG = Peroxisome proliferator-activated receptor γ*; *CEBPA* = *CCAAT/enhancer-binding protein α; FABP4* = f*atty acid binding protein 4; WNT10B = wingless-type MMTV integration site family 10B; ACTB* = *β-actin TOP2B* = *Topoisomerase II-beta*.

There were a total of 48 readings (Cqs) for each of the genes studied in the four muscles and the SAT; those data were analyzed as specified by the nested design (4 animals x 3 samples per tissue x 2 RTs x 2 qPCRs) in order to estimate the contribution of the main sources of variability to the total variation. Mean, minimum, maximum and coefficient of variation (CV) of Cqs obtained for each gene and estimated standard deviations of the different factors that affect to the experiment and total variance are shown in Tables 6–8. The contribution of the processing steps to the variance was also expressed as percentages to facilitate the comprehension of the results (Fig 3).

**Table 6:** Mean, minimum, maximum and coefficient of variation (CV) of Cq values and estimated standard deviations for inter-animal variation, processing steps (Sampling, RT and qPCR) and total variance in muscles *Longissimus thoracis*, *Semitendinosus*, *Masseter* and *Sternomandibularis* for adipogenic markers.

	*LT*^1^				*ST*				*MS*				*SM*			
Item	*PPARG*^2^	*CEBPA*	*FABP4*	*WNT10B*	*PPARG*	*CEBPA*	*FABP4*	*WNT10B*	*PPARG*	*CEBPA*	*FABP4*	*WNT10B*	*PPARG*	*CEBPA*	*FABP4*	*WNT10B*
Mean Cq	26.43	28.80	25.63	32.20	27.34	29.26	27.33	32.29	26.38	28.79	26.72	31.34	27.61	28.99	28.15	31.09
Minimum	23.64	26.21	20.89	29.08	23.09	24.54	17.98	28.62	25.06	25.70	24.11	28.22	25.76	27.17	24.65	28.75
Maximum	28.56	30.91	29.69	39.44	32.69	34.36	34.55	36.35	28.50	31.09	29.91	33.35	30.45	30.84	31.00	35.04
CV (%)	3.87	2.94	7.01	6.89	8.09	7.82	15.94	5.64	3.53	4.15	4.44	3.87	4.65	2.86	5.57	4.66
$\hat{\sigma }$																
Animal	0.40	0.31	0.00	0.28	1.03	1.11	0.00	0.00	0.00	0.00	0.43	0.00	0.80	0.28	0.00	0.66
Sampling	0.49	0.37	1.51	0.69	1.69	1.54	3.92	1.34	0.77	1.05	0.57	0.87	0.42	0.05	1.28	0.71
RT	0.53	0.50	0.96	1.64	0.81	1.25	1.63	0.78	0.54	0.63	0.47	0.54	0.94	0.67	0.54	0.85
qPCR	0.62	0.48	0.54	1.08	0.74	0.41	0.80	0.85	0.29	0.48	0.76	0.56	0.27	0.37	0.70	0.57
Total	1.03	0.84	1.87	2.10	2.26	2.31	4.32	1.77	0.99	1.31	1.14	1.17	1.33	0.82	1.56	1.41

^1^*LT* = *Longissimus thoracis*, *ST* = *Semitendinosus*; *MS* = *Masseter*; and *SM* = *Sternomandibularis*

^*2*^*PPARG = peroxisome proliferator-activated receptor γ*; *CEBPA = CCAAT/enhancer-binding protein α; FABP4 = fatty acid binding protein 4; WNT10B* = *wingless-type MMTV integration site family 10B*.

**Table 7:** Mean, minimum, maximum and coefficient of variation (CV) of Cq values and estimated standard deviations for inter-animal variation, processing steps (Sampling, RT and qPCR) and total variance in muscles *Longissimus thoracis*, *Semitendinosus*, *Masseter* and *Sternomandibularis* for reference genes.

	*LT*^1^		*ST*		*MS*		*SM*	
Item	*ACTB*^2^	*TOP2B*	*ACTB*	*TOP2B*	*ACTB*	*TOP2B*	*ACTB*	*TOP2B*
Mean Cq	22.93	24.32	23.26	24.76	22.12	23.90	22.78	24.70
Minimum	20.13	22.22	19.67	21.91	19.98	21.87	20.51	22.50
Maximum	26.27	28.08	29.94	30.90	24.82	27.74	27.08	28.27
CV (%)	7.06	6.09	10.46	8.91	5.91	5.86	7.44	6.87
$\hat{\sigma }$								
Animal	0.00	0.22	1.35	1.04	0.00	0.00	0.00	0.74
Sampling	0.80	0.60	1.36	1.62	0.72	0.00	0.89	1.08
RT	1.45	1.27	1.83	1.00	1.10	1.49	1.28	1.17
qPCR	0.72	0.48	0.56	0.78	0.52	0.22	0.34	0.19
Total	1.80	1.50	2.71	2.30	1.41	1.51	1.59	1.76

^1^*LT* = *Longissimus thoracis*, *ST* = *Semitendinosus*; *MS* = *Masseter*; and *SM* = *Sternomandibularis*

^2^ *ACTB* = *β-actin; TOP2B* = *Topoisomerase II-beta*.

**Table 8:** Mean, minimum, maximum and coefficient of variation (CV) of Cq values and estimated standard deviations for inter-animal variation, processing steps (Sampling, RT and qPCR) and total variance in subcutaneous adipose tissue.

Item	SAT^1^					
	*PPARG*^2^	*CEBPA*	*FABP4*	*WNT10B*	*ACTB*	*TOP2B*
Mean Cq	23.31	24.31	16.66	30.74	20.89	24.14
Minimum	21.21	21.56	14.72	29.19	18.95	22.93
Maximum	26.42	28.16	19.25	32.40	23.07	25.94
CV (%)	5.92	5.98	7.12	2.47	7.42	3.15
$\hat{\sigma }$						
Animal	1.11	1.12	0.70	0.40	0.15	0.54
Sampling	0.73	0.85	0.55	0.00	0.54	0.33
RT	0.59	0.53	0.77	0.69	0.70	0.49
qPCR	0.10	0.23	0.12	0.28	0.13	0.14
Total	1.47	1.52	1.19	0.84	0.91	0.81

^1^SAT = subcutaneous adipose tissue

^2^ *PPARG = peroxisome proliferator-activated receptor γ*; *CEBPA = CCAAT/enhancer-binding protein α; FABP4 = fatty acid binding protein 4; WNT10B* = wingless-*type MMTV integration site family 10B; ACTB* = *β-actin; TOP2B* = *Topoisomerase II-beta*

The genes *PPARG*, *CEBPA*, and *FABP4*, had Cq values that ranged from 26 to 29 cycles in the four muscles studied, whereas the reference genes, *ACTB* and *TOP2B*, ranged from 22 to 25. *WNT10B* had the lower expression and ranged from 31 to 32 cycles in the muscles. In a similar way, in the SAT the expression of *PPARG*, *CEBPA* and *FABP4* was higher than the expression of *WNT10B*. For all genes, CV values were below 10% except for FABP4 (15.9%) and ACTB (10.5%) in ST muscle (Tables 6 and 7 respectively).

Referring to the variance contributions of the processing steps, the results showed that, in general, the RT represented the higher percentage of the total variance followed by sampling. The standard deviation average values obtained for the sampling and RT steps in the four muscles studied were 34% and 39% respectively and the sum of both represented in average, the 73% of the total variance. On the contrary, qPCR variance was low in most of cases. The muscle *ST* presented the higher total variability, which was associated with the sampling as source of variation. The factor “animal” expressed higher variability in the SAT than in the muscles.

## Discussion

It is widely accepted that IMF is an attribute of economic importance as it influences meat quality. In cattle, IMF content varies greatly between breeds but it also differs within animals and muscles [35]. There are several factors involved in this variability, from rearing conditions, such as feeding [36] or age endpoint, to the relative importance of oxidative and glycolytic metabolism in muscle fibers [37]. Then, studying different muscles could improve the understanding of the IMF development and the factors involved in its accumulation. Therefore, the main objective of this work was to analyze potential differences between different bovine muscles in terms of IMF, collagen and protein content, adipocyte size distribution and gene expression of different key genes involved in the IMF accretion.

Adipose tissue deposition is attributed to either an increase of adipocyte number (hyperplasia), adipocyte volume (hypertrophy) or a combination of both [38–40]. Intensity of hyperplasia and hypertrophy processes are influenced by factors such as genotype, sex, age, feeding regimen, and the individual adipose tissue depot [38, 39, 41]. Then, the study of the size and number of adipocytes might help to elucidate the different contribution of hypertrophy and hyperplasia of adipose cells to lipid accumulation [42] and therefore, to better understand the relationship between those processes and the amount of fat stored in the tissues.

In the current work, adipocytes from *MS* and *SM* muscles, as well as from the SAT, showed a bimodal distribution (Table 3) typically observed for the adipose tissue [43, 44], and characterized for having a small and a large adipocyte population. On the contrary, in *LT* and *ST* muscles a unique adipocyte population of adipocytes, presenting a unimodal size distribution, was observed (Table 2). That difference might be related to the different development stages that can be found in the various adipose tissue depots at a fixed point during the growing process of the animals [45]. From a developmental point of view, IMF is the less mature and the last adipose tissue depot to accumulate fat, and the SAT develops earlier [39, 46], fact that has been observed for various species such as finishing pigs, sheep and cattle [47–49]. Similarly to the fact that different adipose depots develop at different rates, [50] it might be as well that there is a variability in the rate of IMF accretion between muscles, showing different fat development rates. This agree with findings by Roberts *et al*. [51] who indicated that within a single animal not all intramuscular deposits are at the same stage of development. The greater amount of IMF and the bimodal adipocyte size distribution displayed by the *MS* and *SM* muscles could indicate a more active hypertrophy process than in the *LT* and *ST* muscles. A delay in adipocyte hypertrophy can be associated with a lower maturity or delayed fat deposition [51, 52]. Therefore, the results suggest that the IMF develops later in *LT* and *ST* than in the other two muscles studied in the current work, resulting in a delay in the fat deposition in those muscles, as corroborated by the greater amount of fat in MS and SM muscles. On the other hand, as mentioned above, the amount of fat can be influenced as well by the type of fiber. Then the greater amount of IMF content observed in *MS* and *SM* muscles compared to *LT* and *ST* (Table 1) could be related to a higher proportion of oxidative fibers, which contain more phospholipids and triglycerides [4].

In order to investigate if the differences found in the IMF content and the adipocyte size distribution were related to adipocyte differentiation and fatty acid metabolism, the expression of some key adipogenic genes (*PPARG*, *CEBPA*, *FABP4* and *WNT10B)* was analyzed in the four muscles.

*PPARG* and *CEBPA* are central regulators of adipogenesis and act together in the last stages of the adipocyte differentiation program as pleiotropic transcriptional activators of the large group of genes that produce the adipocyte phenotype [53–56]. The expression of *PPARG* and *CEBPA* have been related to IMF content in cattle in previous works and a positive relationship between the expression of these genes and the amount of fat has been found [50, 57]. Other studies nevertheless did not show such relationship [58, 59].

In the present work, the differences in the IMF content and the cell size distribution displayed by the four muscles were not reflected by the gene expression of *PPARG* and *CEBPA* as no statistically significant differences between muscles were observed (Table 4). As mentioned above, other authors also found no concordance between the amount of fat in different adipose tissues and the expression of adipogenic genes such as *PPARG*. For instance, Pickworth *et al*. [59] observed that Angus-Simmental crossbred steers differing in *Longissimus dorsi* muscle fat percentage did not show differential *PPARG* expression. These authors hypothesized that the group of animals with the lower amount of IMF in the *Longissimus* muscle had adipocytes undergoing differentiation whereas the other group might had greater numbers of mature adipocytes, resulting in similar *PPARG* relative expression; they attributed the differences in intramuscular adipose tissue to differences in *PPARG* exhibited at an earlier stage of development.

The lack of differences in gene expression between muscles displaying different adipocyte size distributions and amount of IMF may be then related to the contribution to the total gene expression of the big adipocytes population in *MS* and *SM* muscles, considering that *PPARG* and *CEBPA* are also expressed in mature adipocytes [60]. Besides, Bennet *et al*. [61] postulated that during adipogenesis and after day 3 of differentiation, expression of *CEBPA* and *PPARG* is self-supporting through positive feedback regulation, which might add to the results obtained.

The gene *FABP4* is mainly expressed in the adipocytes [62, 63] and encodes for proteins related to fatty acid uptake, transport and metabolism [64], being involved in fat deposition [65–67]. In spite of young bulls at the end-point having a greater amount of IMF in *MS* and *SM*, and the fact that some observations showed increased *FABP4* expression as IMF developed [59], those muscles had lower *FABP4* expression than *LT*. The reason for this result remains unknown but might be related to the involvement of *FABP4* in the long chain fatty acid accretion and the different nutrient partitioning between organs and tissues that could occur at different points of the development of animals. On the other hand, the accumulation of FABP protein could be as well an indicator of adipocyte number within the muscle tissue [68, 69] and then imply a higher number of adipose cells, albeit probably of smaller size, in LT muscle.

*WNT10B* is known to have a role in blocking adipogenesis, its expression is elevated in preadipocytes and declines upon induction of differentiation [56, 61, 70]; it also seems to be involved in the inhibition of the IMF deposition [71]. Nevertheless, in the present work, and similarly to the case of *PPARG* and *CEBPA*, *WNT10B* gene expression did not present statistical differences between the muscles studied.

Taking into account the results obtained for the study of the gene expression in the four muscles, it was considered of interest to compare the IMF, a late developing adipose tissue, with another that develops early in the life of animals such as the SAT. Then, gene expression of the markers of interest was analyzed and a higher expression of *PPARG*, *CEBPA* and *FABP4* in the SAT than in the four muscles was observed (Table 5). On the contrary, *WNT10B* expression did not differ between muscles and SAT. Differences between IMF and SAT at the molecular level could be expected considering the different characteristics of both adipose depots. The differences found in the expression of *PPARG*, *CEBPA* and *FABP4* are in agreement with the SAT presenting bigger (Table 3), and possibly more adipocytes, than the muscles. Other studies also corroborated these differences between depots [49, 59].

Regarding *WNT10B*, it was previously found that its expression was upregulated in the *Longissimus* muscle in Pirenaica young bulls compared to the SAT [6]. Other authors observed that the expression of *WNT10B* was high in stromal vascular cells (which contains predominantly preadipocytes) and undetectable in mice primary adipocytes, which is consistent with the role of *WNT10B* in maintaining the preadipocytes in an undifferentiated state [61]. Furthermore, when WNT signaling was prevented, preadipocytes underwent enhanced differentiation [70]. The results obtained in the present work might then be indicative of the presence of the population of cells in the pre-adipocyte state in the SAT.

It is known that gene expression data show a high variability, which reduces the ability to detect statistical significance. This fact can be overcome, to some extent, by an appropriate experiment design, which would take into account the preceding steps to the amplification by qPCR, and their contribution to the measurement error. In addition, confounding biological variation and analytical noise in a qPCR assay limit the ability to observe differential expression of treatment groups. Thus, a correct design that minimizes the variability is essential for a better interpretation of the data.

In the present work, the effects of the inter-animal variation and the processing noise of the analytical steps, which for animal tissues are the major factors that contribute to the variability in gene expression quantification, were evaluated. The contribution of the inter-animal variation to the total variance, that represents the biological variation among animals, was the lowest but it was also difficult to estimate with precision due to the small number of animals considered. In order to obtain a more representative results, and focusing on the sources of variability that can be controlled in the analytical steps, it has been highlighted the importance of taking more than a single sample per tissue [72]. In addition, Tichopad *et al*. [12] recommended the use of sample replicates preferentially to any other replicates when working with solid tissues. Based in those previous observations, the design of the present experiment included three different samples per tissue. The results obtained showed that the sampling and RT processing steps had the higher variability, explaining the highest CVs found and representing the 73% of the total variance. The final processing step was qPCR and its contribution to the total variance was consistently lower than the contribution of the other steps. Therefore, qPCR step showed the highest repeatability. The results obtained in the present study are in concordance with the conclusion reached by Tichopad *et al*. [12]. Then, the inclusion of sample and RT replicates in the design of future experiments is recommended preferentially to any other replicates in order to reduce the results variability, while the main reason to run qPCR replicates is to insure against a failed reaction so that the data point is not missed.

To summarize, the results obtained in the present work suggest that the different IMF accretion in the muscles studied might be related to different rates of hyperplasia and hypertrophy. In addition, IMF might develop later in *LT* and *ST* muscles, based on the observations that *MS* and *SM* muscles had greater amount of IMF and adipocytes that followed a bimodal distribution while in the muscles *LT* and *ST*, with less IMF, the adipocyte size distribution was unimodal. That would agree with the fact that the more glycolytic muscle, eg. *Semitendinosus*, have lower levels of IMF [69]. Though, the differences in IMF amount and adipocyte size distribution were not mirrored by the expression of the adipogenic genes studied, result that might be related to the different rates of hyperplasia and hypertrophy and contribution of mature and non-mature adipocytes to the total expression. This was reinforced by the results obtained when studying the SAT, an early developing adipose depot that presents bigger adipocytes and higher number of adipose cells.

Animal and qPCR replicates are usually favored against sample and RT replicates in gene expression studies but the results obtained indicate that the latter are the analytical factors that add higher variability to the gene expression and accordingly, it would be appropriate to include sample and RT replicates in the design of future experiments. However, that implies experiments with bigger number of samples and analysis and, therefore a bigger economic cost. In this respect, and in order to reduce the high processing variability of the sampling and RT steps, various replicates of the samples can be taken and pooled for the RNA extraction, allowing then RT replicates. In the case of studies involving cattle, the substitution of the *LT* muscle by another of lower economic value might be suitable when studying gene expression. As previously said, the expression of the genes related to the IMF deposition (*PPARG*, *CEBPA*, and *WNT10B)* was similar between the muscles studied. Thus, *MS* and *SM* muscles, which had lower variability than *ST* and have a lower economic importance in relation to *LT*, could allow less expensive experimental designs and therefore bigger sample size that could permit the detection of lower relevant differences in gene expression.

## Supporting information

S1 File Material and methods: cDNA synthesis and RT-qPCR. (DOCX)

S1 Table Oligonucleotide sequences and amplicon size of the target genes *peroxisome proliferator-activated receptor γ*, *CCAAT/enhancer-binding protein α*, *fatty acid binding protein 4*, *wingless-type MMTV integration site family 10B*, *β-actin* and *topoisomerase II-beta*. ^1^ Primer direction. F = forward; R = Reverse.^2^ Amplicon Size in base pairs (bp).^3^ *PPARG = peroxisome proliferator-activated receptor γ*; *CEBPA = CCAAT/enhancer-binding protein α; FABP4 = fatty acid binding protein 4; WNT10B* = *wingless-type MMTV integration site family 10B; ACTB* = *β-actin; TOP2B* = *Topoisomerase II-beta*.
(DOCX)

S2 Table Slope, R^2^ and efficiency values for the target genes *peroxisome proliferator-activated receptor γ*, *CCAAT/enhancer-binding protein α*, *fatty acid binding protein 4*, *wingless-type MMTV integration site family 10B*, *β-actin* and *topoisomerase II-beta*. ^1^ Slope of the standard curve.^2^ R^2^ = coefficient of determination of the standard curve^3^ Calculated as [10(-1/Slope)].^4^*PPARG = peroxisome proliferator-activated receptor γ*; *CEBPA = CCAAT/enhancer-binding protein α; FABP4 = fatty acid binding protein 4; WNT10B* = *wingless-type MMTV integration site family 10B; ACTB* = *β-actin; TOP2B* = *Topoisomerase II-beta*.
(DOCX)
