# Supplementary material for: Adiposity and adipogenic gene expression in four different muscles in beef cattle
Source: PLoS One. 2017 Jun 30;12(6):e0179604. doi: 10.1371/journal.pone.0179604 (PMC5493301; doi:10.1371/journal.pone.0179604)
Supplement: S1 File — (DOCX) [file pone.0179604.s001.docx]

**Material and Methods**

**cDNA synthesis and RT-qPCR**

Prior to the cDNA synthesis, the RNA was treated with DNase using RQ1 RNases-Free DNase (Promega Corporation, Madison, WI, USA) as follows: 1 µl of RQ1 RNase-Free DNase 10x Reaction Buffer, 1 µl RQ1 RNase-Free DNase, from 1-8 µl of isolated RNA (final RNA concentration 750 ng) and DEPC water up to 10 µl. Samples were incubated 30 minutes at 37ºC; then 1 µl of RQ1 Stop solution was added and the samples were incubated for 10 minutes at 65ºC.

Singled stranded cDNA was synthetized using PrimeScript RT Reagent (Takara, Japan) preparing a mix in PCR tubes for each sample containing: 2 µl of 5X Buffer, 0.5 µl of RT enzyme mix, 0.5 µl of Oligo dt primer (50µl), 0.5 µl of Randon primers 6 mer (100µM), 500ng of RNA (from 3.5 to 6.5 µl) and DEPC water up to a final volume of 10 µl. The samples were placed in the Gene Amp PRC System 9700 (Applied Biosystems, Foster City, CA, USA) and incubated 15 minutes at 37ºC and 5 seconds at 85ºC.

The expression analysis was performed using a FX96 Touch™ Real-Time PCR Detection System (Bio-Rad, Munich, Germany). The qPCR was conducted preparing a mix in PCR tubes for each sample containing: 5 µl of SYBR Premix Ex Taq (Takara, Japan), 0.5 µl of forward and reverse primers (10 µM), 1 µl of DNase-RNase Free water and DEPC water for a final volume of 7 µl.

The mix was distributed in 96-well clear Multiplate PCR plates, (Bio-Rad, Munich, Germany) and 3 µl of cDNA (1:5 dilution) were added to each well. Then the plate was sealed with Microseal `B´seal Seals (Bio-Rad, Munich, Germany) and placed in the FX96 Touch™ Real-Time PCR Detection System (Bio-Rad, Munich, Germany), which incorporates the Bio-CFX Manger 3.0 program. The cycling conditions of qPCR included a first denaturation at 95°C for 30 seconds and 40 cycles of amplification consisted in a denaturation at 95°C for 5 seconds followed by a hybridization/elongation phase of 30 seconds at 60°C. The final steps of the qPCR-cycling program were 5 seconds at 65ºC and 5 seconds at 95ºC.

Dissociation curves and melting temperatures were examined for the presence of a single RT-qPCR product. The reactions that presented multiple peaks in the dissociation curve were performed again using different primers concentration in order to ensure their specificity. The reactions that presented none melting temperature were considered as failed and those that presented an amplification in the two negative controls (reactions carry out without sample) carried out for each gene in each plate were considered as contaminated. To ensure the reliability of the data, the reactions considered as failed or contaminated were repeated. In addition, since each qPCR reaction was performed by duplicate, those replicates whose Cq value differed more than one unit were repeated.

In order to perform a more detailed analysis, the Cq values were analysed using boxplot diagrams generated with SPSS software V22.0. Cq values above or below the upper or lower quartile respectively were considered as outliers and were studied carefully in order to determine whether they should be eliminated or not. An outlier may be due to the measurement variability or to an error related to the experimental procedures, so in order to identify outliers due to experimental errors, the amplification curves and the melting temperatures were examined as described previously. Therefore, the outliers that corresponded to errors in the experimental procedures were not considered.
