# Supplementary material for: Adiposity and adipogenic gene expression in four different muscles in beef cattle
Source: PLoS One. 2017 Jun 30;12(6):e0179604. doi: 10.1371/journal.pone.0179604 (PMC5493301; doi:10.1371/journal.pone.0179604)
Supplement: S1 Table — 1 Primer direction. F = forward; R = Reverse.2 Amplicon Size in base pairs (bp).3 PPARG = peroxisome proliferator-activated receptor γ; CEBPA = CCAAT/enhancer-binding protein α; FABP4 = fatty acid binding protein 4; WNT10B = wingless-type MMTV integration site family 10B; ACTB = β-actin; TOP2B = Topoisomerase II-beta. (DOCX) [file pone.0179604.s002.docx]

**S1 Table. Oligonucleotide sequences and amplicon size of the target genes *peroxisome proliferator-activated receptor γ,* *CCAAT/enhancer-binding protein α, fatty acid binding protein 4, wingless-type MMTV integration site family 10B, β-actin* and *topoisomerase II-beta.***

| **Gene ID** | **Accession Number** | **Gene** | **Primers^1^** | **bp^2^** | **Source** |
| --- | --- | --- | --- | --- | --- |
| *Peroxisome proliferator-activated receptor γ* | NM_181024 | *PPARG*^3^ | F: GTG AAG TTC AAC GCA CTG GA  R: ATG TCC TCA ATG GGC TTC AC | 113 | [1] |
| *CCAAT/enhancer-binding protein α* | NM_176784 | *CEBPA* | F: TGG ACA AGA ACA GCA ACG AG  R: TTG TCA CTG GTC AGC TCC AG | 130 | [1] |
| *Fatty acid binding protein* | NM_174314.2 | *FABP4* | F: CAT CTT GCT GAA AGC TGC AC  R: ACC CCC ATT CAA ACT GAT GA | 202 | [2] |
| *Wingless-type MMTV integration site family 10B* | XM_005206363 | *WNT10B* | F: AAT GCA AGT GCC ATG GTA CG  R: GAG TTG CGG TTG TGA GTA TCA ATG | 139 | [1] |
| *β-actin* | BC_142413 | *ACTB* | F: CGC CAT GGA TGA TGA TAT TGC  R: AAG CGG CCT TGC ACA TGC | 65 | [2] |
| *Topoisomerase II-beta* | XM_001254709 | *TOP2B* | F: CCG ATG ATG ATG ACG ACA AT  R: TGC TAT GGG AGA TGC TTT GA | 62 | [3] |

^1^ Primer direction. F= forward; R= Reverse.

^2^ Amplicon Size in base pairs (bp).

^3^ *PPARG = peroxisome proliferator-activated receptor γ*; *CEBPA = CCAAT/enhancer-binding protein α; FABP4 = fatty acid binding protein 4; WNT10B* = *wingless-type MMTV integration site family 10B; ACTB* = *β-actin;* *TOP2B* = *Topoisomerase II-beta.*

**Supplementary references**

1. Soret B, Mendizabal JA, Arana A, Alfonso L. Expression of genes involved in adipogenesis and lipid metabolism in subcutaneous adipose tissue and longissimus muscle in low-marbled Pirenaica beef cattle. Animal. 2016;10(12):2018-26. doi: 10.1017/S175173111600118X.

2. Bonnet M, Bernard L, Bes S, Leroux C. Selection of reference genes for quantitative real-time PCR normalisation in adipose tissue, muscle, liver and mammary gland from ruminants. Animal: an international journal of animal bioscience. 2013;7(8):1344-53. doi: 10.1017/S1751731113000475.

3. Waters SM, Kenny DA, Killeen AP, Spellman SA, Fitzgerald A, Hennessy AA, et al. Effect of level of eicosapentaenoic acid on the transcriptional regulation of Delta-9 desaturase using a novel in vitro bovine intramuscular adipocyte cell culture model. Animal: an international journal of animal bioscience. 2009;3(5):718-27. doi: 10.1017/S1751731109004054.
