# Supplementary material for: Adiposity and adipogenic gene expression in four different muscles in beef cattle
Source: PLoS One. 2017 Jun 30;12(6):e0179604. doi: 10.1371/journal.pone.0179604 (PMC5493301; doi:10.1371/journal.pone.0179604)
Supplement: S2 Table — 1 Slope of the standard curve.2 R2 = coefficient of determination of the standard curve3 Calculated as [10(-1/Slope)].4PPARG = peroxisome proliferator-activated receptor γ; CEBPA = CCAAT/enhancer-binding protein α; FABP4 = fatty acid binding protein 4; WNT10B = wingless-type MMTV integration site family 10B; ACTB = β-actin; TOP2B = Topoisomerase II-beta. (DOCX) [file pone.0179604.s003.docx]

**S2 Table. Slope, R^2^ and Efficiency values for the target genes *peroxisome proliferator-activated receptor γ*, *CCAAT/enhancer-binding protein α*, *fatty acid binding protein 4*, *wingless-type MMTV integration site family 10B*, *β-actin* and *topoisomerase II-beta*.**

| **Gene** | **Slope^1^** | **(R^2^)^2^** | **Efficiency^3^** | |
| --- | --- | --- | --- | --- |
| *PPARG^4^* | -3.405 | 0.994 | | 1.967 |
| *CEBPA* | -3.493 | 0.983 | | 1.933 |
| *FABP4* | -3.546 | 0.984 | | 1.914 |
| *WNT10B* | -3.684 | 0.984 | | 1.868 |
| *ACTB* | -3.450 | 0.964 | | 1.948 |
| *TOP2B* | -3.020 | 0.993 | | 2.000 |

^1^ Slope of the standard curve.

^2^ R^2^= coefficient of determination of the standard curve

^3^ Calculated as [10(-1/Slope)].

^4^*PPARG = peroxisome proliferator-activated receptor γ*; *CEBPA = CCAAT/enhancer-binding protein α; FABP4 = fatty acid binding protein 4; WNT10B* = *wingless-type MMTV integration site family 10B; ACTB* = *β-actin;* *TOP2B* = *Topoisomerase II-beta.*
